# Observer-Based Adaptive Sliding Mode Compensation Position-Tracking Control for Drilling Tool Attitude Adjustment

**DOI:** 10.3390/s24082404

**Published:** 2024-04-09

**Authors:** Jinheng Gu, Xunqi Wang, Haifeng Yan, Chao Tan, Lei Si, Zhongbin Wang

**Affiliations:** 1 School of Mechatronic Engineering, China University of Mining and Technology, Xuzhou 221116, China; gujinheng@126.com (J.G.); ts22050178p31@cumt.edu.cn (X.W.); yanhaifeng@cumt.edu.cn (H.Y.); tccadcumt@126.com (C.T.); lei.si@cumt.edu.cn (L.S.); 2 Jiangsu Key Laboratory of Mine Mechanical and Electrical Equipment, China University of Mining and Technology, Xuzhou 221116, China

**Keywords:** observer-based controller, adaptive sliding mode control, position-tracking control, drilling tool attitude adjustment system, coal mine drilling robot for rockburst prevention

## Abstract

This study develops an adaptive sliding mode control approach for a drilling tool attitude adjustment system, aiming at solving the problems of model uncertainties and insufficient ability of disturbance suppression during the regulation behavior. To further improve the performance of the position-tracking loop in terms of response time, tracking accuracy, and robustness, a state observer based on an improved radial basis function is designed to approximate the model uncertainties, a valve dead-zone compensate controller is used to reduce control deviation, an adaptive sliding mode controller is designed to improve the position-tracking precision and attenuate sliding mode chattering. Finally, simulation and experimental results are carried out to verify the observability of the model uncertainties and position-tracking errors of the drilling tool attitude adjustment system, which can effectively improve the position-tracking performance and robustness of the drilling tool attitude adjustment system.

## 1. Introduction

Coal mine rockburst has become one of the most serious dynamic disasters for deep coal mining in China, which has a significant impact on the safe mining of coal [1,2,3]. The borehole pressure relief method has become an effective way in coal mine rockburst prevention and control, and adjusting the drilling tool to align with the pressure relief hole is the precondition for achieving an effective borehole [4]. Then, the high precision adjustment control of the drilling tool platform is a prevalent choice for the intelligent borehole, which is a construct drilling tool attitude adjustment system (DTAAS). In the DTAAS, a commonly employed configuration involves front and rear lifting hydraulic cylinders with precise position control. The efficiency and reliable operation of the lifting hydraulic cylinders directly affect the lifting position and pitch angle of the DTAAS. However, the position-tracking accuracy and stability of the DTAAS are decreased because of the model uncertainties and external disturbances [5,6]. Considering the different position-tracking conditions, the control methods of the DTAAS are still in exploration.

Many studies have contributed to developing control techniques in the field of the electrohydraulic control system, which provides a feasible way to achieve accurate position control for the DTAAS, e.g., robust position control [7,8], active disturbance rejection control [9,10,11], predictive control [12,13], sliding mode control (SMC) [14,15,16,17], and adaptive control [18,19,20,21]. Among these methods, SMC is an effective control method for decreasing the disturbances in the nonlinear system because of its strong robustness and quick response [22,23]. For instance, a sliding mode controller with a high-gain observer was designed to improve the system response characteristics of the electrohydraulic system [24,25]. A sliding mode controller with disturbances rejection for the electrohydraulic asymmetric cylinder system is designed to achieve accurate position control and attenuate the disturbances, including model uncertainties, and external disturbance [26]. A hybrid sliding mode controller is designed with load disturbance suppression, and the chattering phenomenon is reduced [27]. Although the SMC method is effective in different application areas, relevant application of the SMC method in the DTAAS is still scarce. Moreover, the conventional SMC method, which lacks the estimation of model uncertainties and disturbances, is not suitable for the position-tracking control of the DTAAS. Therefore, a new position-tracking control scheme urgently needs to be proposed to improve the system response characteristics for the DTAAS.

In the field of electrohydraulic valve control systems, ubiquitous and difficultly resolved problems of the input nonlinearities (i.e., dead zone, saturation) have attracted the attention of researchers. A dead-zone compensator is incorporated into the controller design, which is used to reduce the influence of nonlinear input error [28]. Considering the input dead zone, an integrated position synchronization control strategy for dual-electrohydraulic actuators has been developed [29]. Additionally, the effect of the dead zone can be compensated effectively via adaptive law, which can improve tracking performance [30]. The input dead-zone model can be linearized and an adaptive cooperative neural dynamic surface control is proposed [31]. The input dead zone can be approximated by a non-affine smooth function, which is used to improve the tracking control performance [32]. However, there is still no provided way with which to handle the input dead-zone nonlinearity in the DTAAS. Moreover, the mixture effects of input dead-zone and system uncertainties in the DTAAS may bring more challenges for position-tracking control.

In designing a controller for the DTAAS, when the position loop experiences external disturbances, such as external loads, and parameter mismatches, designing a state observer for the DTAAS position-tracking controller becomes crucial to ensure accurate position control and anti-disturbance capability. An SMC-based method has been designed to ensure system performance and an extended state observer for the disturbances and uncertainties suppression is proposed for the electro-hydraulic actuator [33]. Furthermore, the state and disturbance estimator on a hydraulic actuator are designed to improve the system response characteristics [34]. Consequently, the model uncertainties and disturbance require estimation and compensation within the position-tracking control to eliminate the tracking deviations in the regulation motion of the DTAAS. Addressing these problems involves leveraging observers to estimate the disturbances and improve control performance. Many studies have indicated that disturbance observers can realize the desired estimation performance. For example, a disturbance observer-based control has been proposed to achieve high-accuracy tracking for a hydraulic system [35], and a disturbance observer-based backstepping tracking control has been designed to compensate for the uncertainties and estimation errors to guarantee the response performance for an electrohydraulic actuator system [36], and it has been applied to various systems [37,38,39,40]. Therefore, a state observer is proposed to attenuate the influence of the disturbances and further enhance the anti-disturbance capability of the DTAAS. In the design process of the controller for the DTAAS, the estimation values of the uncertainty items are added in the control law, the regulated parameters vary with the system state, and the regulation process is adaptively regulated, i.e., adaptive adjustment.

According to the previous discussion, a sliding mode compensation control method for the DTAAS is proposed. The main contributions are summarized as follows:

(1) The proportional directional valve dead-zone characteristics are analyzed for the DTAAS position loop to achieve the faster response property, thereby improving the position control efficiency of the DTAAS;

(2) A state observer based on an improved radial basis function is designed to observe the unknown nonlinear disturbance including model uncertainties and external disturbances. Meanwhile, the adaptive control law is used to compensate the SMC to further improve the accuracy of the DTAAS;

(3) The position-tracking and anti-disturbance performance of the proposed method are verified by the simulation and experimental results.

The remaining paper is organized as follows. Section 2 shows the mathematical model of the DTAAS with the model uncertainties, and the valve dead-zone characteristics are given. In Section 3, the state observer and adaptive sliding mode controller are designed, and the position-tracking control strategy is proposed. Simulation and experiments are conducted to verify the effectiveness of the proposed method in Section 4. Section 5 concludes this paper.

## 2. Drilling Tool Attitude Adjustment System and Theoretical Foundations

### 2.1. System Description

**Figure 1:**
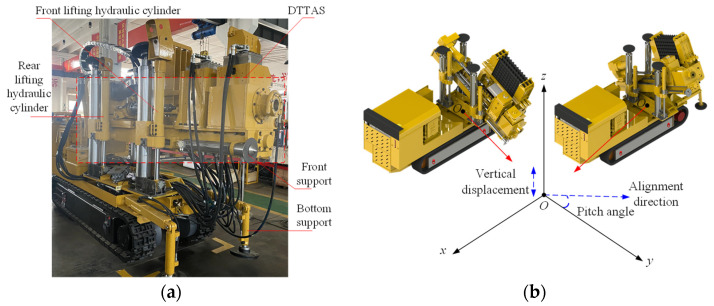
The schematic diagram of a DTAAS: (**a**) the main components in a DTAAS, (**b**) schematic diagram for adjusting the vertical displacement and pitch angle.

The drilling tool attitude adjustment system of the coal mine drilling robot for rockburst prevention has a direct impact on the alignment accuracy of the coal mine borehole, which is determined by the displacement regulation of the front and rear lifting hydraulic cylinders. For the DTAAS, the lifting position and pitch angle are controlled by the displacement of the front and rear lifting hydraulic cylinders. Therefore, the alignment accuracy of the coal mine borehole mainly depends on the position control accuracy of the front and rear lifting hydraulic cylinders. These front and rear lifting hydraulic cylinders are coordinated and controlled by the electrohydraulic control system, to achieve stable and precise attitude adjustment, and the schematic diagram of a DTAAS of the coal mine drilling robot for rockburst prevention is shown in Figure 1.

**Figure 2:**
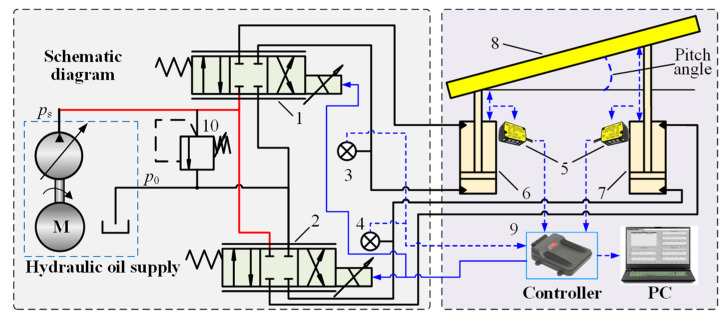
Drilling tool attitude adjustment system principle with electrohydraulic proportional control. Notes: 1,2—electrohydraulic proportional directional valve; 3,4—pressure sensor; 5—displacement sensor; 6—rear lifting hydraulic cylinder; 7—front lifting hydraulic cylinder; 8—simplified model of the DTAAS; 9—controller; 10—safety relief valve.

Referring to Figure 1, the lifting displacement and pitch angle of the DTAAS are regulated by the position control of the front and rear lifting hydraulic cylinders, using the displacement sensors as feedback input, the controller can compute the adjustment value and transmit these control signal to the electrohydraulic proportion valves, which has different output response characteristics of the drilling tool attitude adjustment. Therefore, a displacement sensor information continuous acquisition and electrohydraulic proportional control system configuration based on a DTAAS as shown in Figure 2, is proposed. The front and rear lifting hydraulic cylinders are separately controlled by proportional directional valves. The controller and the host computer are utilized to acquire the displacement signal, the pitch angle signal, the output electrohydraulic proportional directional valve control signal and control parameters debugging.

Taking the forward direction of the coal mine drilling robot for rockburst prevention as *x* direction, the vertical movement direction of the drilling tool platform as *z* direction, the direction perpendicular to the supporting platform of the coal mine drilling robot for rockburst prevention as *y* direction, the center projection point between the front and rear lifting hydraulic cylinders as the origin point *O*. The pitch angle can be calculated as:

(1) $$\theta =\arctan\frac{x_{L1}-x_{L2}}{l_{1}}$$

where *θ* is the pitch angle, *l*_1_ is the vertical projection distance between the front and rear lifting hydraulic cylinders, pitch angle, *x*_L1_ and *x*_L2_ are the displacement values of the front and rear lifting hydraulic cylinders, respectively.

### 2.2. Mathematical Model of the Drilling Tool Attitude Adjustment System

For the DTAAS, the front and rear lifting hydraulic cylinders have the same motion function to support the regulation of the drilling tool platform. As the position-tracking control condition for enhancing controllability, the designed control strategy must generate sufficient dynamic tracking performance to adapt to hydraulic cylinder adjustment [41,42]. The vertical position and pitch angle of the DTAAS changes with the extension and retraction of the front and rear lifting hydraulic cylinders, which are controlled by the electrohydraulic proportional directional valves. Correspondingly, the mathematical model of the DTAAS can be described as:

(2) $$\left\{\begin{array}{l} p_{L}=\frac{p_{1}A_{1}-p_{2}A_{2}}{A_{1}}=p_{1}-np_{2}\;x_{v}\geq 0\\ p_{L}=\frac{p_{2}A_{2}-p_{1}A_{1}}{A_{2}}=p_{2}-\frac{p_{1}}{n}\;x_{v}<0\\ q_{L}=q_{1}=q_{2}/n\\ n=A_{2}/A_{1} \end{array}\right.$$

where *p*_L_ is the load pressure; *A*_1_ and *A*_2_ represent the effective action area of rodless and rod chambers, respectively; *p*_1_ and *p*_2_ represent the hydraulic pressure of rodless and rod chambers, respectively; *x*_v_ represents the spool displacement of the proportional directional valve.

The proportional directional valve serves as the control mechanism for the displacement regulation of the DTAAS, the nonlinear dynamic relationship between hydraulic oil flow rate and the spool displacement can be described as:

(3) $$\left\{\begin{array}{cc} \left.\begin{array}{l} p_{1}=\frac{n^{3}p_{s}+m^{2}p_{L}}{m^{2}+n^{3}},q_{1}=mC_{\mathrm{sv}}w_{1}x_{v}\sqrt{\frac{2}{\rho }\cdot \frac{\left(p_{s}-p_{L}\right)}{m^{2}+n^{3}}}\\ p_{2}=\frac{n^{2}\left(p_{s}-p_{L}\right)}{m^{2}+n^{3}},q_{2}=nC_{\mathrm{sv}}w_{3}x_{v}\sqrt{\frac{2}{\rho }\cdot \frac{\left(p_{s}-p_{L}\right)}{m^{2}+n^{3}}} \end{array}\right\} & x_{v}\geq 0\\ \left.\begin{array}{l} p_{1}=\frac{m^{2}n\left(p_{s}-p_{L}\right)}{m^{2}+n^{3}},q_{1}=mC_{\mathrm{sv}}w_{2}x_{v}\sqrt{\frac{2}{\rho }\cdot \frac{n\left(p_{s}-p_{L}\right)}{m^{2}+n^{3}}}\\ p_{2}=\frac{m^{2}p_{s}{+n}^{3}p_{L}}{m^{2}+n^{3}},q_{2}=nC_{\mathrm{sv}}w_{4}x_{v}\sqrt{\frac{2}{\rho }\cdot \frac{n\left(p_{s}-p_{L}\right)}{m^{2}+n^{3}}} \end{array}\right\} & x_{v}<0 \end{array}\right.$$

where m = *w*_3_/*w*_1_ = *w*_4_/*w*_2_ is the ratio of the spool valve area gradient corresponding to the rod and rodless chambers of the hydraulic cylinder.

Combing Equations (2) and (3), and then:

(4) $$\left\{\begin{array}{ll} \Delta q_{L}=K_{\mathrm{qv}}\Delta x_{v}-K_{\mathrm{cv}}\Delta p_{L} & \\ \begin{array}{l} K_{\mathrm{qv}}=mC_{\mathrm{sv}}w_{1}\sqrt{\frac{2}{\rho }\cdot \frac{\left(p_{s}-p_{L}\right)}{m^{2}+n^{3}}}\\ K_{\mathrm{cv}}=mC_{\mathrm{sv}}w_{1}x_{v}\sqrt{\frac{1}{2\rho \left(p_{s}-p_{L}\right)\left(m^{2}+n^{3}\right)}} \end{array} & x_{v}\geq 0\\ \begin{array}{l} K_{\mathrm{qv}}=mC_{\mathrm{sv}}w_{2}\sqrt{\frac{2}{\rho }\cdot \frac{n\left(p_{s}-p_{L}\right)}{m^{2}+n^{3}}}\\ K_{\mathrm{cv}}=mC_{\mathrm{sv}}w_{2}x_{v}\sqrt{\frac{1}{2\rho n\left(p_{s}-p_{L}\right)\left(m^{2}+n^{3}\right)}} \end{array} & x_{v}<0 \end{array}\right.$$

where *K*_qv_, *K*_cv_ represent the flow and flow-pressure gain coefficient, respectively; *C*_sv_ represents the valve throttling coefficient.

Additionally, the relationship between hydraulic oil flow rate and the load pressure can be described as:

(5) $$\left\{\begin{array}{l} \left.\begin{array}{l} q_{L}=A_{1}\frac{dx_{L}}{\mathrm{dt}}+\frac{V_{t}}{2\beta_{e}\left(1+n^{2}\right)}\frac{dp_{L}}{\mathrm{dt}}+\mu_{1}p_{L}+\lambda_{1}C_{\mathrm{ip}}p_{s}\\ \lambda_{1}=\frac{n^{2}\left(n^{2}-1\right)}{\left(1+n^{2}\right)\left(m^{2}+n^{3}\right)},\;\mu_{1}=\frac{\left(1+n\right)\left(m^{2}+n^{2}\right)}{\left(1+n^{2}\right)\left(m^{2}+n^{3}\right)}C_{\mathrm{ip}}+\frac{C_{\mathrm{ii}}}{1+n^{2}} \end{array}\right\}x_{v}>0\\ \left.\begin{array}{l} q_{L}=A_{2}\frac{dx_{L}}{\mathrm{ndt}}+\frac{V_{t}n}{2\beta_{e}\left(1+n^{2}\right)}\frac{dp_{L}}{\mathrm{dt}}+\mu_{2}p_{L}+\lambda_{2}C_{\mathrm{ip}}p_{s}\\ \lambda_{2}=\frac{m^{2}\left(1-n^{2}\right)}{\left(1+n^{2}\right)\left(m^{2}+n^{3}\right)},\mu_{2}=\frac{\left(1+n\right)\left(m^{2}+n^{2}\right)C_{\mathrm{ip}}}{\left(1+n^{2}\right)\left(m^{2}+n^{3}\right)}+\frac{nC_{\mathrm{ii}}}{1+n^{2}} \end{array}\right\}x_{v}<0 \end{array}\right.$$

So that, Equation (6) can be expressed as:

(6) $$\left\{\begin{array}{l} \begin{array}{ll} \begin{array}{ll} A_{1}{\dot{x}}_{L} & =-\frac{V_{t}}{2\beta_{e}\left(1+n^{2}\right)}{\dot{p}}_{L}-\mu_{1}p_{L}-\lambda_{1}C_{\mathrm{ip}}p_{s}\\ & +x_{v}mC_{\mathrm{SV}}w_{1}\sqrt{\;\frac{2}{\rho \left(m^{2}+n^{3}\right)}\left(p_{s}-p_{L}\right)} \end{array} & x_{v}>0 \end{array}\\ \begin{array}{ll} \begin{array}{ll} A_{2}{\dot{x}}_{L} & =-\frac{V_{t}n^{2}}{2\beta_{e}\left(1+n^{2}\right)}{\dot{p}}_{L}-\mu_{2}np_{L}-\lambda_{2}nC_{\mathrm{ip}}p_{s}\\ & +x_{v}\mathrm{mn}C_{\mathrm{SV}}w_{2}\sqrt{\frac{2}{\rho }\cdot \frac{n\left(p_{s}-p_{L}\right)}{m^{2}+n^{3}}} \end{array} & x_{v}<0 \end{array} \end{array}\right.$$

The displacement of the drilling tool platform is driven by the hydraulic cylinder piston rod to extension and retraction, and there is unmeasurable friction between the piston rod and the hydraulic cylinder. Defining the system comprehensive uncertainty items as ∆*F*_d_, including friction, external interference and other items that are difficult to model. The system load balance equation can be expressed as:

(7) $$A_{1}p_{L}=m_{s}{\ddot{x}}_{L}+k_{s}x_{L}+\Delta F_{d}$$

Considering the dead-zone characteristics of the proportional directional valve, the relationship between spool displacement and the control signal *u*_v_ can be described as:

(8) $$x_{v}=f\left(k_{v}\right)u_{v}=\left\{\begin{array}{cc} x_{\mathrm{vmin}} & u_{v}<u_{\min}\\ k_{v}u_{v} & u_{\min}<u_{v}<u_{\max}\\ x_{\mathrm{vmax}} & u_{\max}<u_{v} \end{array}\right.$$

Considering the parameter adaptation of Equation (6), the dynamic equation of the DTAAS can be rewritten as:

(9) $$\begin{array}{l} {\dot{x}}_{L}=f(p_{L},t)+g(x_{v},t)u_{v}\\ \left\{\begin{array}{l} \left.\begin{array}{l} f(p_{L},t)=\frac{1}{A_{1}}(-\frac{V_{t}}{2\beta_{e}\left(1+n^{2}\right)}{\dot{p}}_{L}-\mu_{1}p_{L}-\lambda_{1}C_{\mathrm{ip}}p_{s})\\ g(x_{v},t)=\frac{1}{A_{1}}\left(mC_{\mathrm{SV}}w_{1}\sqrt{\;\frac{2\left(p_{s}-p_{L}\right)}{\rho \left(m^{2}+n^{3}\right)}}\right)f\left(k_{v}\right) \end{array}\right\}x_{v}>0\\ \left.\begin{array}{l} f(p_{L},t)=\frac{1}{A_{2}}(-\frac{V_{t}n^{2}}{2\beta_{e}\left(1+n^{2}\right)}{\dot{p}}_{L}-\mu_{2}np_{L}-\lambda_{1}nC_{\mathrm{ip}}p_{s})\\ g(x_{v},t)=\frac{1}{A_{2}}\left(\mathrm{mn}C_{\mathrm{SV}}w_{2}\sqrt{\;\frac{2n\left(p_{s}-p_{L}\right)}{\rho \left(m^{2}+n^{3}\right)}}\right)f\left(k_{v}\right) \end{array}\right\}\;x_{v}<0 \end{array}\right. \end{array}$$

### 2.3. Dead-Zone Characteristics of the Proportional Directional Valve

**Figure 3:**
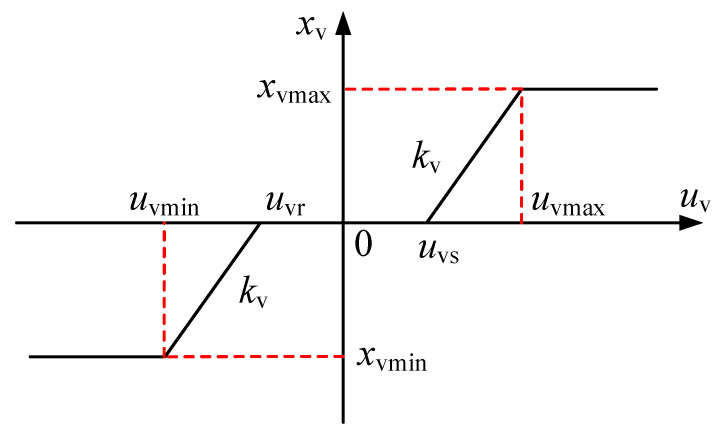
Dead-zone nonlinear model of the proportional directional valve.

For the operation control of the DTAAS, the hydraulic oil is controlled by the proportional directional valve to enter the hydraulic cylinder, making the hydraulic cylinder extend or retract. Owing to the coulomb friction and production process intrinsic characteristics, the proportional directional valve has a certain amount of repeated coverage at the valve port when the valve spool in the middle position, resulting in the movement of the proportional valve spool to be not controlled by input signals with a certain range. For this reason, a dead zone is produced for the proportional directional valve, which causes the hydraulic cylinder cannot timely respond to the control signal. During the position adjustment operations of the DTAAS, the lifting position and pitch angle need to constantly change, which causes the valve spool to pass through the zero position and the system to be unable to accurately respond and regulate. To solve this problem and investigate the dead-zone characteristics, the dead-zone nonlinear model for the proportional directional valve is established, as shown in Figure 3.

The dead-zone nonlinear model includes a neutral dead zone and two linear parts [43]. Considering the differences in the proportional directional valve, the dead-zone nonlinear model can be expressed as:

(10) $$x_{v}=\left\{\begin{array}{cc} x_{\mathrm{vmin}} & u_{v}<u_{\min}\\ k_{v}\left(u_{v}-f\left(u_{v}\right)\right) & u_{\min}<u_{v}<u_{\max}\\ x_{\mathrm{vmax}} & u_{\max}<u_{v} \end{array}\right.$$

(11) $$f\left(u_{v}\right)=\left\{\begin{array}{cc} u_{\mathrm{vr}} & u_{v}<u_{\mathrm{vr}}\\ u_{v} & u_{\mathrm{vr}}<u_{v}<u_{\mathrm{vs}}\\ u_{\mathrm{vs}} & u_{\mathrm{vs}}\leq u_{v} \end{array}\right.$$

where *x*_vmin_, *x*_vmax_ represent the lower and upper limit values, respectively; *k*_v_ represent the valve amplification coefficient; *u*_vr_, *u*_vs_ represent the threshold values for entering and leaving the dead zone, respectively, which determine the width of the dead zone. Additionally, Equation (8) can be rewritten as Equation (10).

Based on the dead-zone nonlinear model, the control performance of the proportional directional valve is greatly limited by the dead-zone characteristics, which is important to compensate for the dead-zone nonlinear characteristics. In view of the dead-zone characteristics, the inverse function model of the valve dead zone is designed to compensate for the dead-zone nonlinear characteristics. Assuming the *u*_vr_, *u*_vs_ are bounded and can be obtained, i.e., *u*_vrmin_ < *u*_vr_ < *u*_vrmax_ < 0, 0 < *u*_vsmin_ < *u*_vs_ < *u*_vsmax_; *u*_vg_ = [*u*_vr_, *u*_vs_]^T^, the output of valve dead-zone compensate controller can be designed as:

(12) $$u_{\mathrm{vg}}={{\hat{u}}_{\mathrm{vg}}}^{T}\phi_{v}=\left(1-\phi \left(u_{v}\right)\right){\hat{u}}_{\mathrm{vr}}+\phi \left(u_{v}\right){\hat{u}}_{\mathrm{vs}}$$

(13) $$\phi \left(u_{v}\right)=\left\{\begin{array}{cc} 0 & u_{v}<0\\ 1 & u_{v}\geq 0 \end{array}\right.$$

(14) $$\phi_{v}={\left[\begin{array}{cc} \phi \left(u_{v}\right), & 1-\phi \left(u_{v}\right) \end{array}\right]}^{T}$$

where ${\hat{u}}_{\mathrm{vg}}$ is the estimation value of the $u_{\mathrm{vg}}$.

## 3. Design of Adaptive Sliding Mode Compensation Position-tracking Controller

### 3.1. Design of the State Observer Based on Improved RBF Neural Network

In order to improve the control speed, accuracy and robustness capability of the DTAAS of the coal mine drilling robot for rockburst prevention, a state observer based on the improved radial basis function (RBF) neural network is designed to effectively identify the uncertain system model, which can be used to approximate the uncertainty item of ∆*F*_d_. For this purpose, assuming the *x* is the input value of the RBF neural network, *ε* is the approximate error, *W*_1_ = [*w*_11_, *w*_12_, *w*_13_, …, *w*_1n_]^T^ is the connection weights among network nodes, and the output can be described as:

(15) $$f_{1}\left(x\right)=W_{1}^{T}\;Sn_{1}\left(x\right)+\varepsilon_{1}$$

where *f*_1_(*x*) represents the uncertainty item; *Sn*_1_(*x*) = [*sn*_11_, *sn*_12_, *sn*_13_, …, *sn*_1n_]^T^ represents the radial basis function vector of the RBF neural network.

The radial basis function can be described as:

(16) $$Sn_{1j}\left(x\right)=\exp\left(\frac{{\left\|x-\sigma_{1j}\right\|}_{2}}{2b_{1j}^{2}}\right)$$

where *σ*_1*j*_(*x*) = [*σ*_11*j*_, *σ*_12*j*_, *σ*_13*j*_]^T^ and *b*_1*j*_ represent the center and width of the *j*th node, respectively; $\sigma_{1j}\in R,i=1,2,3,j=1,2,\cdots ,n$.

By adjusting the number of the hidden layer neurons and connection weights, the RBF network can effectively approximate the uncertainty item, and the number of neurons directly affects the learning speed and training accuracy. An inappropriate number of neurons will easily reduce the learning speed and recognition efficiency. In view of this, the dynamically adjusting the number of the hidden layer neurons based on the deviation between the data sample and the central sample is proposed in this paper. Assuming the *x^1i^* is the *i*th sample, $r_{l_{1}}$ is the node center with the minimum distance from the sample, $l_{1}=\arg\underset{j}{\min}{\left\|x^{1i}-\sigma_{1j}\right\|}_{2}$. Additionally, the redundant neuron nodes are merged based on the node center distance among neurons. $\sigma_{1p}$ and $\sigma_{1q}$ are the any two neuron node centers, when ${\left\|\sigma_{1p}-\sigma_{1q}\right\|}_{2}<\omega_{1}$, the two neurons merge into one neuron, and the $\omega_{1}$ is the coefficient of determination. The merged neuron node center is described as:

(17) $${\bar{\sigma }}_{1p}=\frac{\sigma_{1p}+\sigma_{1q}}{2}$$

When the improved RBF network is used to identify the uncertainty items of the DTAAS, the *W*_1_ is unknown, assuming the ${\hat{W}}_{1}$ is the estimation value of the *W*_1_, the error can be described as:

(18) $${\tilde{W}}_{1}=W_{1}-{\hat{W}}_{1}$$

the estimation value of the uncertainty items can be described as:

(19) $${\hat{f}}_{1}\left(x\right)={\hat{W}}_{1}^{T}\;Sn_{1}\left(x\right)$$

### 3.2. Design of the Conventional Sliding Mode Controller

For the nonlinear dynamic system of the DTAAS, an integral sliding surface is designed to improve the control precision of the position-tracking control.

(20) $$s_{c}=e_{c1}+k_{c1}\int_{0}^{t}e_{c1}dt$$

where *s_c_* is the integral sliding surface; *k_c_*_1_ > 0 is the coefficient of the sliding mode control; *e_c_*_1_ is the displacement error of the controlled lifting hydraulic cylinder.

(21) $$e_{c1}=x_{L}-x_{L\exp}$$

where *x*_Lexp_ and *x*_L_ represent the expected and actual displacement, respectively.

Taking the derivative of the Equation (20), and then:

(22) $${\dot{s}}_{c}={\dot{e}}_{c1}+k_{c1}e_{c1}$$

Based on this, the sliding mode reaching law based on the saturation function can be designed as:

(23) $${\dot{s}}_{c}=-\varepsilon_{c1}sat\left(s_{c}\right)-k_{c2}s_{c}$$

where *ε_c_*_1_ > 1, and *k_c_*_2_ > 0.

If the Lyapunov function is employed as:

(24) $$V_{1}=\frac{1}{2}s_{c}^{2}$$

And then, the derivative of the Equation (24) is described as:

(25) $$\begin{array}{l} {\dot{V}}_{1}={\dot{s}}_{c}s_{c}=s_{c}\left({\dot{e}}_{c1}+k_{c1}e_{c1}\right)=s_{c}\left({\dot{x}}_{L}-{\dot{x}}_{L\exp}+k_{c1}e_{c1}\right)\\ =s_{c}\left(f(p_{L},t)+g(x_{v},t)u_{v}-{\dot{x}}_{L\exp}+k_{c1}e_{c1}\right) \end{array}$$

The sliding mode controller can be designed as:

(26) $$u_{v}=\frac{1}{g(x_{v},t)}\left(-f(p_{L},t)+{\dot{x}}_{L\exp}-k_{c1}e_{c1}-k_{c3}s_{c}\right)$$

where *k_c_*_3_ > 0.

Combining the Equations (25) and (26), ${\dot{V}}_{1}<0$ represent the system is stable.

### 3.3. Design of the Adaptive Sliding Mode Controller

To further improve the control accuracy and robustness of the position-tracking control for the DTAAS, an adaptive sliding mode controller is designed, and the integral sliding surface is designed as:

(27) $$s_{a}=e_{a1}+k_{a1}\int_{0}^{t}e_{a1}dt+{\dot{e}}_{a1}$$

where *s_a_* is the integral sliding surface; *k_a_*_1_ > 0 is the coefficient of the sliding mode control; *e_a_*_1_ is the displacement error of the controlled lifting hydraulic cylinder.

(28) $$e_{a1}=x_{L}-x_{L\exp}$$

where *x*_Lexp_ and *x*_L_ represent the expected and actual displacement, respectively.

Taking the derivative of the Equation (27), and then:

(29) $${\dot{s}}_{a}={\dot{e}}_{a1}+k_{a1}e_{a1}+{\ddot{e}}_{a1}$$

The sliding mode reaching law based on the saturation function can be designed as:

(30) $${\dot{s}}_{a}=-\varepsilon_{a1}{e_{a1}}^{{\left|s_{a}\right|}^{\alpha }}sat\left(s_{a}\right)-k_{a2}{\left|s_{a}\right|}^{\beta }s_{a}$$

where *ε_a_*_1_ > 1, *k_a_*_2_ > 0, 0 < *α* < 1, 0 < *β* < 1.

If the Lyapunov function is employed as:

(31) $$V_{2}=\frac{1}{2}s_{a}^{2}$$

And then, the derivative of the Equation (31) is described as:

(32) $$\begin{array}{l} {\dot{V}}_{2}={\dot{s}}_{a}s_{a}=s_{a}\left({\dot{e}}_{a1}+k_{a1}e_{a1}+{\ddot{e}}_{a1}\right)=s_{a}\left({\dot{x}}_{L}-{\dot{x}}_{L\exp}+k_{a1}e_{a1}+{\ddot{e}}_{a1}\right)\\ =s_{a}\left(f(p_{L},t)+g(x_{v},t)u_{v}-{\dot{x}}_{L\exp}+k_{a1}e_{a1}+{\ddot{e}}_{a1}\right) \end{array}$$

The adaptive sliding mode controller can be designed as:

(33) $$u_{v}=\frac{1}{g(x_{v},t)}\left(-f(p_{L},t)+{\dot{x}}_{L\exp}-k_{a1}e_{a1}-{\ddot{e}}_{a1}-k_{a3}s_{a}\right)$$

where *k_a_*_3_ > 0.

Combining the Equations (32) and (33), ${\dot{V}}_{2}<0$ represent the system is stable.

Based on the designed integral sliding mode surface of Equation (27) and sliding mode reaching law of Equations (30), the adaptive sliding mode control law can be designed, and Equation (33) can be rewritten as:

(34) $$u_{v}=\frac{1}{g(x_{v},t)}\left({\hat{f}}_{1}\left(x\right)-f(p_{L},t)+{\dot{x}}_{L\exp}-k_{a1}e_{a1}-{\ddot{e}}_{a1}-k_{a3}s_{a}-\varepsilon_{a1}{e_{a1}}^{{\left|s_{a}\right|}^{\alpha }}sat\left(s_{a}\right)-k_{a2}{\left|s_{a}\right|}^{\beta }s_{a}\right)$$

The adaptive estimation of the connection weights *W*_2_ in the function of $f(p_{L},t)$ can be designed as:

(35) $${\dot{\hat{W}}}_{2}=\frac{1}{\Delta F_{d}}s_{a}Sn_{2}\left(x\right)$$

If the Lyapunov function is employed as:

(36) $$V_{3}=\frac{1}{2}s_{a}^{2}+\frac{1}{2}\Delta F_{d}{\tilde{W}}_{2}^{T}{\tilde{W}}_{2}$$

Based on Equations (34) and (35), the derivative of the Equation (36) is then described as:

(37) $$\begin{array}{l} {\dot{V}}_{3}={\dot{s}}_{a}s_{a}=s_{a}\left({\dot{e}}_{a1}+k_{a1}e_{a1}+{\ddot{e}}_{a1}\right)-\Delta F_{d}{\tilde{W}}_{2}^{T}{\dot{\hat{W}}}_{2}\\ =s_{a}\left({\dot{x}}_{L}-{\dot{x}}_{L\exp}+k_{a1}e_{a1}+{\ddot{e}}_{a1}\right)-\Delta F_{d}{\tilde{W}}_{2}^{T}\dot{\hat{W}}\\ =s_{a}\left({\hat{f}}_{1}\left(x\right)+f(p_{L},t)+g(x_{v},t)u_{v}-{\dot{x}}_{L\exp}+k_{a1}e_{a1}+{\ddot{e}}_{a1}\right)-\Delta F_{d}{\tilde{W}}_{2}^{T}\dot{\hat{W}}\\ =s_{a}\left({\tilde{W}}_{2}^{T}Sn_{2}\left(x\right)-k_{a3}s_{a}-\varepsilon_{a1}{e_{a1}}^{{\left|s_{a}\right|}^{\alpha }}sat\left(s_{a}\right)-k_{a2}{\left|s_{a}\right|}^{\beta }s_{a}\right)-{\tilde{W}}_{2}^{T}s_{a}Sn_{2}\left(x\right)\\ =s_{a}\left(-k_{a3}s_{a}-\varepsilon_{a1}{e_{a1}}^{{\left|s_{a}\right|}^{\alpha }}sat\left(s_{a}\right)-k_{a2}{\left|s_{a}\right|}^{\beta }s_{a}\right) \end{array}$$

According to Lyapunov stability theory, ${\dot{V}}_{3}<0$ represent the system is stable under the designed controller.

### 3.4. The Proposed Position-Tracking Control Strategy

**Figure 4:**
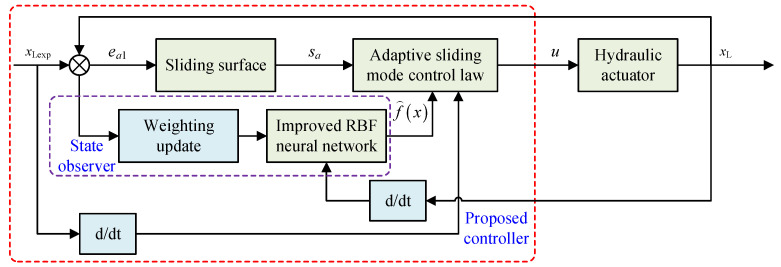
Schematic diagram of the position-tracking control scheme for the DTAAS.

To cope with the nonlinear motion characteristics of the DTAAS, the regulator often cooperates with dynamic operating conditions to execute the control output. Displacement sensors and electrohydraulic proportional directional valves are available to obtain the real-time feedback signal and output the expected operating trajectory, and the designed controller used for calculation is fast running. Finally, the proposed position-tracking control scheme with the proposed method is shown in Figure 4. The proposed scheme improves the control accuracy and robustness of the DTAAS. Moreover, the sliding mode chattering phenomenon is also effectively suppressed.

## 4. Results and Discussion

### 4.1. Main Parameters

In order to verify the control accuracy and effectiveness of the proposed method, comparative simulation and experimental results of the proportion integration differentiation (PID), sliding mode control (SMC), and the adaptive sliding mode control (ASMC) with the state observer-based on improved RBF neural network methods are given. A simulation model is established based on AMESim and MATLAB/Simulink, and their main parameters are listed in Table 1. The controller parameters of the PID are configured as *k*_P_ = 0.19, *k*_I_ = 0.15, *k*_D_ = 0.003; the controller parameters of the SMC are configured as *ε_c_*_1_ = 2, *k_c_*_1_ = 0.8, *k_c_*_2_ = 5, *k_c_*_3_ = 5; the control parameters of the ASMC are configured as *ε_a_*_1_ = 2, *k_a_*_1_ = 0.8, *k_a_*_2_ = 5, *k_a_*_3_ = 5, α = 0.6, *β* = 0.6.

### 4.2. Simulation Results and Analysis

#### 4.2.1. System Response Performance and Analysis

**Figure 5:**
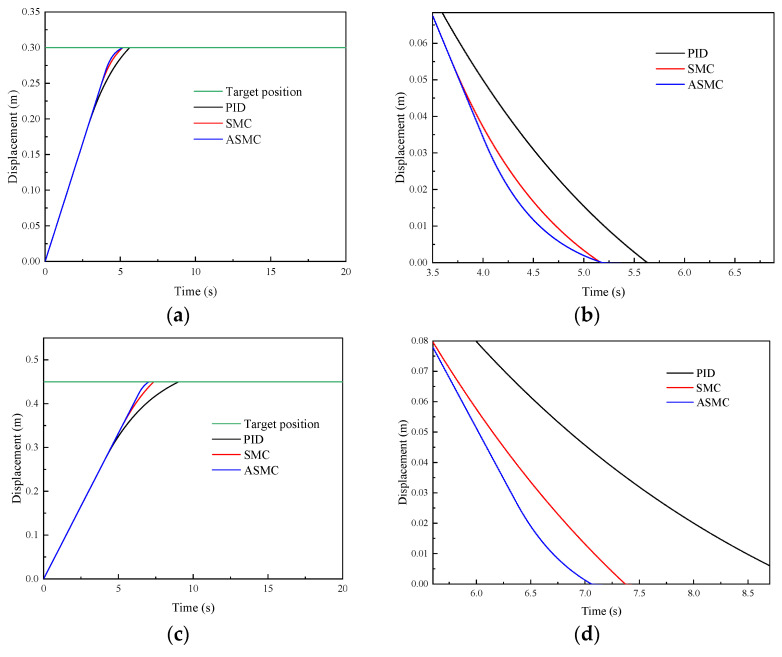
Comparative simulation results of the PID, SMC and ASMC methods in tracking step signal: (**a**) Simulation results of the front lifting hydraulic cylinder with different methods; (**b**) Simulation results of the position-tracking error for the front lifting hydraulic cylinder; (**c**) Simulation results of the rear lifting hydraulic cylinder with different methods; (**d**) Simulation results of the position-tracking error for the rear lifting hydraulic cylinder.

**Figure 6:**
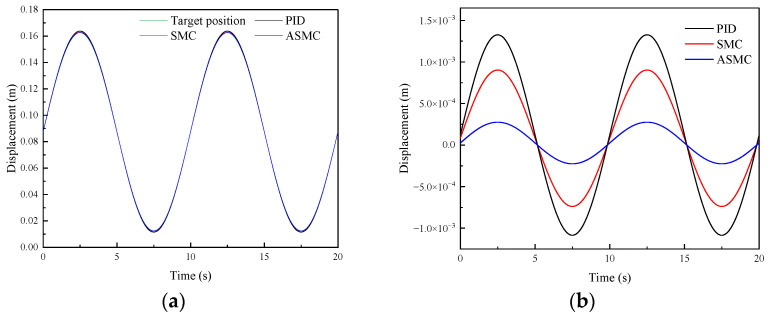

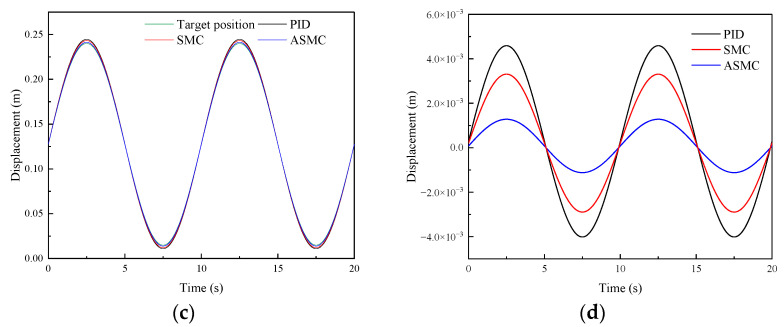
Comparative simulation results of the PID, SMC and ASMC methods in tracking sinusoidal signal: (**a**) Simulation results of the front lifting hydraulic cylinder with different methods; (**b**) Simulation results of the position-tracking errors for the front lifting hydraulic cylinder; (**c**) Simulation results of the rear lifting hydraulic cylinder with different methods; (**d**) Simulation results of the position-tracking errors for the rear lifting hydraulic cylinder.

To analyze the position-tracking performance of the proposed ASMC method, considering the comparative results of the reference tracking on other controllers [44,45], the comparative results are given in this paper. Figure 5 and Figure 6, respectively, show the comparative simulation results of the PID, SMC and ASMC methods in tracking step signal and sine signal. Figure 5 shows the comparative simulation results of different methods in tracking step signals. Figure 5a shows that the settling time of the PID, SMC and ASMC is 5.67 s, 5.25 s, and 5.18 s, respectively. The SMC has a better time response characteristic than the PID, and no significant difference compared with the ASMC. Figure 5b shows that the SMC controller is better than the PID controller in the position-tracking average absolute error (28.34 mm), the position-tracking average absolute error of the SMC is 18.67 mm. The position-tracking average absolute error of the ASMC is 13.38 mm, which is 52.78%, 28.33% less than the PID and SMC. For the rear lifting hydraulic cylinder, Figure 5c shows that the settling time of the PID, SMC and ASMC is 9.05 s, 7.37 s, and 7.06 s, respectively. Figure 5d shows that the SMC controller is better than the PID controller in the position-tracking average absolute error (35.62 mm), the position-tracking average absolute error of the SMC is 20.39 mm. The position-tracking average absolute error of the ASMC is 16.17 mm, which is 54.60%, 20.69% less than the PID and SMC.

Afterward, Figure 6 shows the comparative simulation results of different methods in tracking sinusoidal signals. Figure 6a shows that the SMC has a better time response characteristic than the PID, and no significant difference compared with the ASMC. Figure 6b shows that the SMC controller is better than the PID controller in the position-tracking average absolute error (4.34 mm), the position-tracking average absolute error of the SMC is 2.68 mm. The position-tracking average absolute error of the ASMC is 2.06 mm, which is 52.53%, 23.13% less than the PID and SMC. For the rear lifting hydraulic cylinder, Figure 6c shows that the response times of the PID, SMC and ASMC are fast. Figure 6d shows that the SMC controller is better than the PID controller in the position-tracking average absolute error (6.27 mm), the position-tracking average absolute error of the SMC is 3.42 mm. The position-tracking average absolute error of the ASMC is 2.63 mm, which is 58.05%, 23.09% less than the PID and SMC. From these results, the ASMC has better system response characteristics when position-tracking control under the step and sinusoidal signals.

#### 4.2.2. Model Uncertainties and Analysis

**Figure 7:**
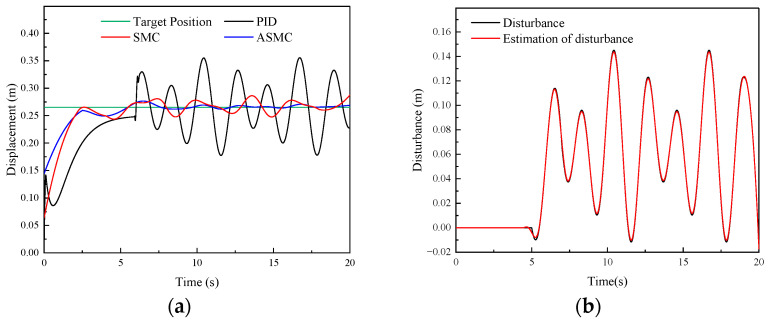

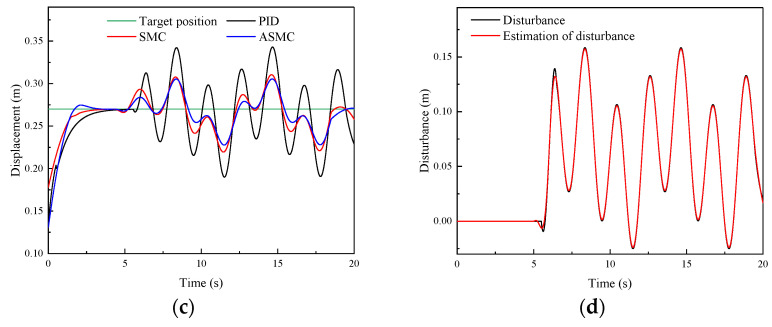
Comparative simulation results of the PID, SMC and ASMC methods in tracking step signal and adding a disturbance signal: (**a**) Simulation results of the front lifting hydraulic cylinder with different methods; (**b**) Observed disturbance by the ASMC for the front lifting hydraulic cylinder; (**c**) Simulation results of the rear lifting hydraulic cylinder with different methods; (**d**) Observed disturbance by the ASMC for the rear lifting hydraulic cylinder.

During the adjustment process of the position-tracking control, DTAAS may suffer from model uncertainties, which will cause position-tracking deviation and disturbances. For this purpose, it is necessary to conduct the control performance analysis under disturbances in order to verify the robustness. Therefore, Figure 7 shows the comparative simulation results of different methods in tracking step signals and adding a disturbance signal. Figure 7a shows the response characteristics curve of the PID, SMC and ASMC, and the settling time is 5.52 s, 4.64 s, and 4.38 s, respectively. The SMC has a better disturbance suppression characteristic than the PID, but it is inferior to the ASMC. Figure 7a shows that the SMC controller is better than the PID controller in the position-tracking fluctuation, and the maximum absolute error of the PID is 89.95 mm, the position-tracking maximum absolute error of the SMC is 44.27 mm. The position-tracking maximum absolute error of the ASMC is 31.44 mm, which is 65.04%, 28.98% less than the PID and SMC. For the rear lifting hydraulic cylinder, Figure 7c shows that the settling time and the maximum absolute error of the PID are 4.31 s, and 72.15 mm, respectively, which is inferior to the 3.91 s, 40.47 mm of the SMC. Then, the settling time and the maximum absolute error of the ASMC are 3.74 s, 34.48 mm, respectively, which are 50.82%, 14.80% less than the PID and SMC in suppressing the disturbance. Furthermore, Figure 7b,d shows that the ASMC can effectively observe the disturbance. From these results, the designed ASMC can compensate for the position-tracking control and reduce the influence of the disturbance, which can indicate that the proposed method has a strong robustness to suppress the disturbance caused by the model uncertainties.

### 4.3. Experimental Results and Analysis

#### 4.3.1. Experimental Platform of the DTAAS

**Figure 8:**
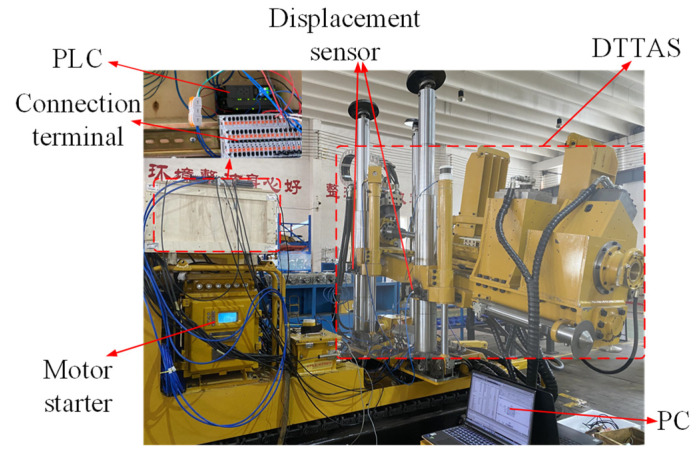
The experimental platform of the DTAAS.

The physical prototype is constructed to validate the designed controller for the DTAAS, and the experimental platform is shown in Figure 8. The designed controller was conducted using PLC 1511C included displacement sensor information acquisition, electrohydraulic proportional directional valve signal output, control method processing, and the continuous sampling frequency of the position-tracking loop is 1 kHz. To further study the practicability and effectiveness of the designed controller for the DTAAS, including position-tracking and anti-disturbance, performance tests were carried out.

#### 4.3.2. Position-Tracking Performance Verification

**Figure 9:**
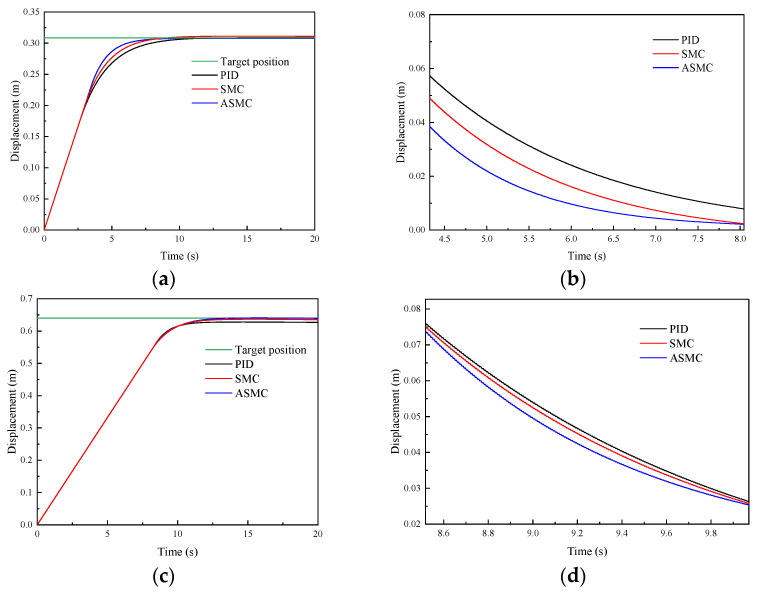
Comparative experimental results of the PID, SMC and ASMC methods in tracking a given position signal: (**a**) Experimental results of the front lifting hydraulic cylinder with different methods; (**b**) Experimental results of the position-tracking errors for the front lifting hydraulic cylinder; (**c**) Experimental results of the rear lifting hydraulic cylinder with different methods; (**d**) Experimental results of the position-tracking errors for the rear lifting hydraulic cylinder.

To verify the position-tracking performance of the proposed ASMC method for the DTAAS, Figure 9 shows the comparative experimental results of position tracking of the PID, SMC and ASMC methods under a given target position signal. Figure 9a shows that the settling time of the PID, SMC and ASMC is 9.87 s, 8.64 s, and 8.13 s, respectively. Figure 9b shows that the SMC controller is better than the PID controller in the position-tracking average absolute error (42.22 mm), the position-tracking average absolute error of the SMC is 28.67 mm. The position-tracking average absolute error of the ASMC is 20.98 mm, which is 50.30%, 26.82% less than the PID and SMC. For the rear lifting hydraulic cylinder, Figure 9c shows that the settling time of the PID, SMC and ASMC is 13.14 s, 12.76 s, and 12.33 s, respectively. Figure 9d shows that the SMC controller is better than the PID controller in the position-tracking average absolute error (56.18 mm), the position-tracking average absolute error of the SMC is 36.83 mm. The position-tracking average absolute error of the ASMC is 26.94 mm, which is 52.04%, 26.85% less than the PID and SMC. Then, the ASMC can further reduce the position-tracking error. Therefore, the proposed ASMC can further improve the position-tracking performance robustness.

#### 4.3.3. Anti-Disturbance Performance Verification

**Figure 10:**
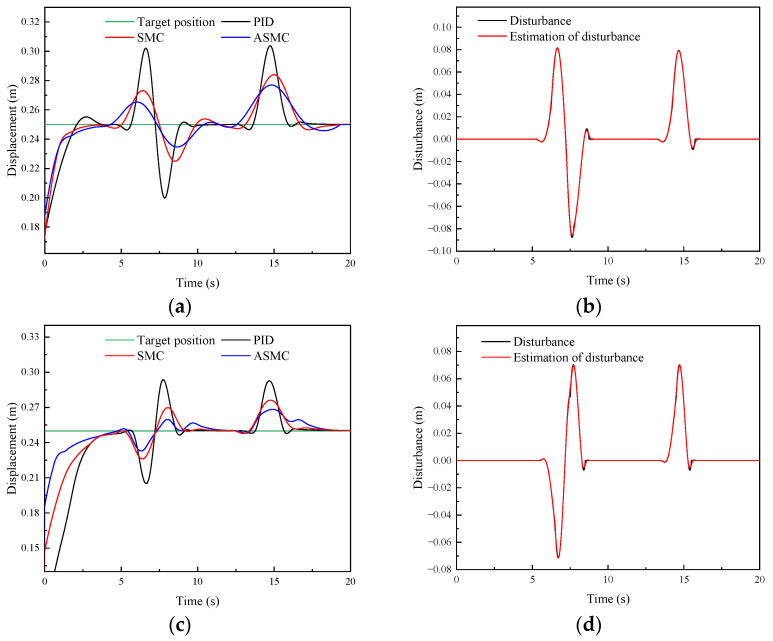
Comparative experimental results of the PID, SMC and ASMC methods in tracking a given target position signal and adding a position disturbance: (**a**) Experimental results of the front lifting hydraulic cylinder with different methods; (**b**) Observed disturbance by the ASMC for the front lifting hydraulic cylinder; (**c**) Experimental results of the rear lifting hydraulic cylinder with different methods; (**d**) Observed disturbance by the ASMC for the rear lifting hydraulic cylinder.

To verify the anti-disturbance performance of the proposed ASMC method for the DTAAS, Figure 10 shows the comparative experimental results of position tracking of the PID, SMC and ASMC methods under a given target position signal and adding a position disturbance. Figure 10a shows the response characteristics curve of the PID, SMC and ASMC, and the settling time is 4.29 s, 3.73 s, and 3.61 s, respectively. The position-tracking maximum absolute error of the SMC is 33.87 mm. With the PID, the maximum absolute error is 53.54 mm. Additionally, the position-tracking maximum absolute error of the ASMC is 26.95 mm, which is 49.66%, 20.43% less than the PID and SMC. Figure 10c shows that the settling time and the maximum absolute error of the PID are 4.23 s, 43.57 mm, respectively, which is inferior to the 3.81 s, 26.18 mm of the SMC. Then, the settling time and the maximum absolute error of the ASMC are 3.79 s, 18.36 mm, respectively, which are 57.86%, 29.87% less than the PID and SMC in suppressing the disturbance. Furthermore, Figure 10b,d show that the ASMC can effectively observe the disturbance and compensate for the position-tracking control. Therefore, it can be indicated that the proposed ASMC has stronger anti-disturbance than the PID and SMC, which can effectively enhance the anti-disturbance capability of the DTAAS.

## 5. Conclusions

A feasible and effective position-tracking control scheme for the DTAAS using the ASMC is proposed. Targeting the large position-tracking deviation and poor anti-disturbance of the DTAAS, an ASMC using a state observer was proposed to achieve rapid response and disturbance suppress performance. Then, the improved RBF is applied to further approximate the uncertainties. Additionally, the sliding mode chattering is also attenuated via the ASMC. Furthermore, simulation and experimental results show that the proposed method can obtain better response characteristics and stronger robustness than the comparison methods. Meanwhile, the disturbance observation and estimation performance of the ASMC is verified by the comparative simulation and experimental results of the ASMC under different position-tracking conditions. In the future, the proposed method could also be employed to position-tracking control in other electrohydraulic systems, such as the hydraulic propulsion system of the shield machine, multi-hydraulic cylinders synchronous position control, etc. Furthermore, an advanced nonlinear position control method could be explored in subsequent research, along with an advanced compensation to reduce delay in the execution and to improve the regulation performance of the electrohydraulic control system.

## Figures and Tables

**Table 1:** Main parameters of the DTAAS.

Symbols	Characteristics	Values
*C*_sv_	Throttling coefficient	0.65
*C*_ip_	Internal leakage coefficient	8.2 × 10^−5^ m^3^/(s∙MPa)
*K*_qv_	Flow gain	1.5 × 10^−10^ m^3^/(s∙MPa)
*K*_cv_	Flow-pressure gain	4.6 × 10^−5^ m^3^/(s∙MPa)
*x*_L_	Maximum stroke	0.8 m
*ρ*	Hydraulic oil density	900 kg/m^3^
*β*e	bulk modulus	1.7 × 10^9^ Pa

## Data Availability

The data are contained within the article.
